# Term-tissue specific models for prediction of gene ontology biological processes using transcriptional profiles of aging in drosophila melanogaster

**DOI:** 10.1186/1471-2105-9-129

**Published:** 2008-02-28

**Authors:** Wensheng Zhang, Sige Zou, Jiuzhou Song

**Affiliations:** 1 Department of Animal and Avian Science, University of Maryland, College park, MD 20742 USA; 2 Bioinformatics Unit, Branch of Research Resources, National Institute on Aging, NIH, Baltimore, Maryland, 21224, USA; 3 Laboratory of Experimental Gerontology, National Institute on Aging, Baltimore, MD 21224, USA

## Abstract

**Background:**

Predictive classification on the base of gene expression profiles appeared recently as an attractive strategy for identifying the biological functions of genes. Gene Ontology (GO) provides a valuable source of knowledge for model training and validation. The increasing collection of microarray data represents a valuable source for generating functional hypotheses of uncharacterized genes.

**Results:**

This study focused on using support vector machines (SVM) to predict GO biological processes from individual or multiple-tissue transcriptional profiles of aging in Drosophila melanogaster. Ten-fold cross validation was implemented to evaluate the prediction. One-tail Fisher's exact test was conducted on each cross validation and multiple testing was addressed using BH FDR procedure. The results showed that, of the 148 pursued GO biological processes, fifteen terms each had at least one model with FDR-adjusted p-value (Adj.p) <0.05 and six had the values between 0.05 and 0.25. Furthermore, all these models had the prediction sensitivity (SN) over 30% and specificity (SP) over 80%.

**Conclusion:**

We proposed the concept of term-tissue specific models indicating the fact that the major part of the optimized prediction models was trained from individual tissue data. Furthermore, we observed that the memberships of the genes involved in all the three pursued children biological processes on mitochondrial electron transport could be predicted from the transcriptional profiles of aging (Adj.p < 0.01). This finding may be important in biology because the genes of mitochondria play a critical role in the longevity of C. elegans and D. melanogaster.

## Background

Assigning function to new genes and understanding the potential unknown biological roles of annotated genes are among the main goals in current biology [1-3]. Microarray technology allows thousands of transcripts to be measured simultaneously on a single slide[4]. Genes coding the same function are likely regulated in the manner of coordination. Predictive classification on the base of gene expression profiles appeared recently as an attractive strategy for identifying the biological functions of genes [3,5,6]. While the major part of DNA microarray datasets were not purposely generated for this objective, they can be useful in generating hypotheses about the functional involvement of genes, especially for those with significant expression patterns across the experimental conditions or over a time span.

Gene Ontology (GO) is a well-known database and a standard terminology for describing functions of gene and gene products across species [7,8]. It provides a valuable resource of knowledge for model training and validation in generating the hypotheses about the functional involvement of unknown genes. In the database, each annotated gene is associated with one or multiple terms of biological processes, molecular functions, and/or cellular components. It is very useful to classify genes based on GO terms for extracting useful information from high throughput gene expression analysis. Recently, a rule-based supervised learning method for the prediction of GO biological processes from temporal gene expression data was developed and validated with a data set describing the transcript levels of genes during the first 24 h of the serum response in serum-starved human fibroblasts [6].

In this paper, we used temporal and spatial transcriptional profiles to predict GO biological processes in Drosophila melanogaster [9]. The gene expression datasets were generated from seven tissues representing nervous, muscular, digestive, renal, reproductive, and storage systems, and measured at five age points. We proposed the concept of term-tissue specific models indicating the fact that the major part of the optimized prediction models was trained from individual tissue data.

## Results

### Predictable biological processes

For each GO biological process (term) containing at least 4 annotated genes (of our list), eight classification models were trained using support vector machines (SVM) with the seven datasets of the individual tissues or the combined dataset. Ten-fold cross validation was implemented to evaluate the prediction. Following, one-tail Fisher's exact test was conducted on each cross validation and multiple testing was addressed using BH FDR procedure [10]. The results showed that, of the 148 pursued GO biological processes, fifteen terms each had at least one model with FDR-adjusted p-value (Adj.p) <0.05 and six had the values between 0.05 and 0.25. Furthermore, all these models had prediction sensitivity (SN) over 30% and specificity (SP) over 80%. The enrichment of functional connections was quite apparent among these 21 terms. For clarity and conveniences, they were grouped by the functional connections (Table 1) and called as "predictable terms", hereafter. The first group included electronic transport (GO: 0006188) and its 3 children terms on mitochondrial electron transport (GO: 0006120, GO: 0006122, GO: 0006123). The second group consisted of lipid metabolic process (GO: 0006629) and one of its major children terms, fatty acid acyl-CoA metabolic process (GO: 0006637). The third group contained three transport terms, namely extracellular transport (GO: 0006858), carbohydrates transport (GO: 0008643), and lipid transport (GO: 0006869). Furthermore, the broad term carbohydrate metabolic process (GO: 0005975) and one of its direct children terms, tricarboxylic acid cycle (GO: 0006099), had low adjusted p-values. Other main predictable terms included chitin metabolic process (GO: 0006030), mitosis (GO: 0007067), and transcription (GO: 0006350). Among the numerous terms related to cellular protein metabolism, only two highly specific terms, namely, ubiquitin-deoendent protein catabolic process (GO: 0006511) and protein deubiquitination (GO:0016579), were among the list.

**Table 1:** Top predictable GO biological processes identified using SVM and tenfold validation with the data of individual tissues or the combined data

**Term**	**Name**	**Model**^a^	**TN**	**FP**	**FN**	**TP**	**Adj.P**^c^
GO:0006118	electron transport	CM	1128	100	56	26	1.35-E06
GO:0006122	mitochondrial electron transport, ubiquinol to cytochrome	CM	1263	41	0	6	1.66-E06
GO:0006120	mitochondrial electron transport, NADH to ubiquinone	CM	1227	59	6	18	1.12-E11
GO:0006123	mitochondrial electron transport, cytochrome c to oxygen	CM	1259	42	5	4	6.78-E03
GO:0006629	lipid metabolic process	Brain	1004	245	41	20	2.01-E01
GO:0006637	acyl-CoA metabolic process	Gut	1044	259	2	5	1.05-E01
GO:0006858	extracellular transport	Fat	1105	169	21	15	2.43-E03
GO:0008643	carbohydrate transport	Fat	1167	126	9	8	5.33-E03
GO:0006869	lipid transport	Gut	1051	241	7	11	5.07-E03
GO:0005975	carbohydrate metabolic process	Fat	1140	115	38	17	6.40-E04
GO:0006099	tricarboxylic acid cycle	Testis	1218	75	7	10	6.34-E04
GO:0006511	ubiquitin -deoendent protein catabolic process	Testis	1075	219	7	9	1.69-E02
GO:0016579	protein deubiquitination	Testis	1043	258	2	7	1.36-E02
GO:0006030	chitin metabolic process	CM	1187	108	6	9	1.22-E04
GO:0051189	prosthetic group metabolic process	MT	1136	157	10	7	6.73-E02
GO:0007067	mitosis	MT	1082	213	8	7	1.31-E01
GO:0006350	transcription	Fat	1203	94	9	4	2.01-E02
GO:0007031	peroxisome organization and biogenesis	Gut	1146	156	3	5	2.53-E02
GO:0006625	protein targeting to perosisome	Gut	1129	174	2	5	2.42-E02
GO:0007498	Mesoderm development	MT	1077	223	5	5	2.03-E01
GO:0015986	ATP synthesis coupled proton transport	DTM	1224	73	9	4	1.11-E01

^a ^The tissues (or organs) generating the data used for model training and validation where MT and DTM represent malpighian tubule, and dorsal thoracic muscle, respectively, and CM represents combined data. ^b ^SN and SP represent sensitivity and specificity, respectively. ^c ^FDR-adjusted p-value.

**Figure 1:**
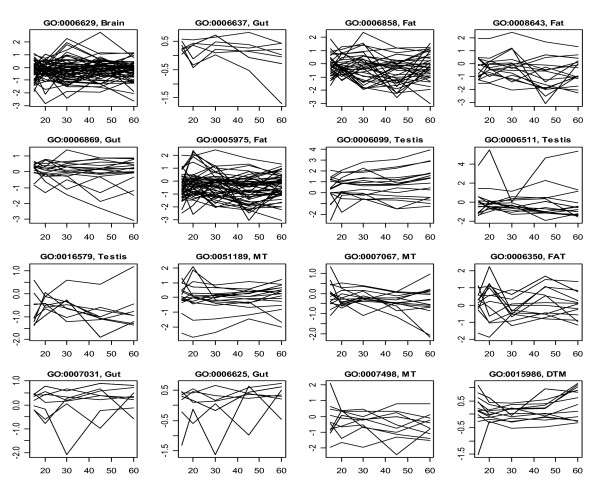
**Transcriptional profiles of genes involved in 16 GO biological processes predictable with term-tissue specific models**. The plot names indicate the IDs of the GO terms and the tissues from which the top prediction models were generated, and the plots were based on the averages of the duplicated measurements at each age points for the corresponding tissues. MT and DTM represent malpighian tubule, and dorsal thoracic muscle, respectively.

**Figure 2:**
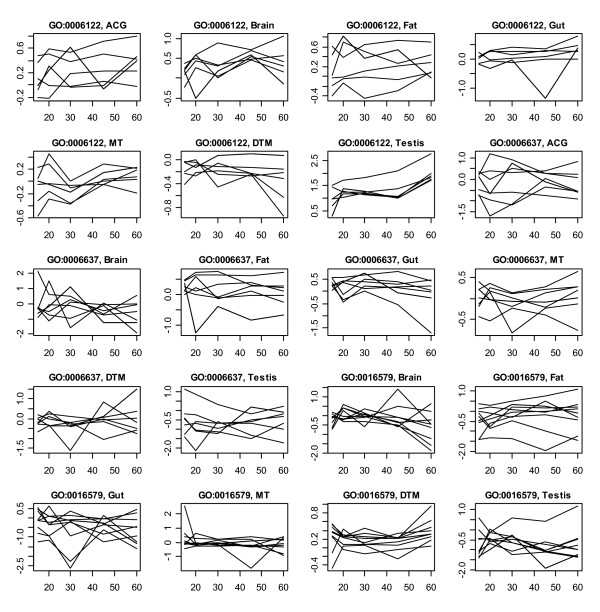
**Tissue-specific transcriptional profiles of genes involved in 3 predictable GO biological processes**. The plots were based on the averages of the duplicated measurements at each age points for the corresponding tissues. ACG, MT and DTM represent accessory gland, malpighian tubule, and dorsal thoracic muscle, respectively.

Genes with different functions can share the similar expression pattern across the aging process, especially when the time points of measurement are relatively sparse such as in the case of our data. As the results, a meaningful prediction needs a high proportion of true positives among the predicted positives. Therefore, in selecting predictable GO terms, we set a relatively higher limit for SP, but this was on the cost of decreasing the criterion for SN. The most terms listed in Table 1 have SN at the levels from 30–75%. Another reason for the lower SN was that, even for these predictable biological processes, only partial member genes were regulated in the manner of coordination (Figure 1). One exception was GO: 0006122, of which all the member genes had the similar expression pattern in testis (Figure 2) and, consequently, the SN was 100%.

### Term-tissue specific models

The prediction of gene function is based on the common pattern shared by the member genes in a specific biological process. It is well known that the regulation of gene expression varies extensively among tissues [11]. In this study, we found that the ageing-related co-regulation patterns of genes involved in a biological process may be tissue-specific. That is, the coordination models for the same biological process may be spatially inconsistent. As indicated in Table 1, the data sources generating the top prediction models varied with the different GO biological processes. This means that, for predicting the potential involvement of an uncharacterized gene in a specific biological process, we need to use the data sourced from an appropriate tissue, although it is possible that the combined dataset will lead to better classification such as in the cases of the terms on mitochondrial electron transport (Table 1). For example, Acyl-CoA metabolic process had its model from gut representing digestive system, and protein deubiquitination had its model from testis representing reproductive system. The results for these two GO terms were listed in Table 2. The first two processes are children terms under lipid metabolic processes. Their two top favorite models could be trained from the data of fat tissue and gut, or from the data of gut and fat tissue, respectively. The difference was that the data from brain and muscle could generate prediction models for fatty acid metabolic but not for the parent term, lipid metabolic process. The third, protein deubiquitination, is a highly-specific GO term under cellular protein metabolic process. For this term, models from muscle and testis had the same SN but different SPs.

**Table 2:** Illustration of term-tissue specific prediction models ^a^

**Term**	**Model**	**SN (%)**	**SP (%)**
GO:0006637	ACG	0	99.9
GO:0006637	Brain	0	92
GO:0006637	Fat	57.1	79.4
GO:0006637	Gut	71.4	80.1
GO:0006637	DT	0	99.3
GO:0006637	DTM	0	100
GO:0006637	Testis	0	99.2
GO:0006637	CM	0	100
			
GO:0016579	ACG	0	98.9
GO:0016579	Brain	11.1	97.8
GO:0016579	Fat	0	99.9
GO:0016579	Gut	0	100
GO:0016579	DT	0	100
GO:0016579	DTM	77.8	66.4
GO:0016579	Testis	77.8	80.
GO:0016579	CM	0	100

^a ^Model, SN and SP are sensitivity and specificity, respectively.

Term-tissue specific models were also characterized by the fact that, even the membership of genes in a GO term may be individually predicted from the datasets of two or more tissues, the expression profiling of these genes varied with the tissues, in general (Figure 2). For example, the function of genes in GO: 0006122 could be predicted both from the expression in brain and testis, but the temporal patterns over ages were largely different. That is, the genes involved in the biological process were seemly regulated with different coordination models in different tissues. At present, we do not know if these different tissue-specified coordination models could be complementary in increasing the prediction accuracy. The current study showed that the simple combination of the data from the 7 tissues had limited advantages. Our further work will be focused on developing more effective algorithm on data integration.

### Hidden gene expression pattern of aging

The microarray experiments generating the data analyzed in this study adopted a reference design with the reference samples made from the flies of 3 days old. Certainly, with the measures between 15 and 60 days, the potential transcriptional patterns of aging formed during 3–15 days could not be detected by significant tests using a linear models [12,13] or non-parameter methods [14-17] if the inference about the intercept (of the statistical model) was ignored. However, these hidden patterns may represent a valuable information source for the functional prediction of new genes. As shown in Figure 1, the profiles of the member genes of many predictable GO terms were distinguished from others with the deviation off the zero line rather than the patterns across the time span of 15–60 days. For example, peroxisome organization and biogenesis (GO: 0007031) was characterized with a nearly flat expression profile approximately at the levels of 0.5.

In order to show the importance of the hidden patterns formed during 3–15 days in establishing prediction models, we conducted a statistical analysis of all 1308 genes with a linear model containing age and tissue as the fixed factors on the measures between 15 and 60 days. Listed in Table 3 are the p-values of the fixed effects and the interaction for the member genes of two predictable biological processes. GO: 0006122 had all its six genes correctly predicted in the cross validation (as shown in Table 1) with the classification models trained from the data of testis or the combined dataset, but only two genes had significant age effect at p < 0.01 and no age-tissue interaction was found for the 6 genes. Similarly, GO: 00016579 had 5 of the 7 member genes predicted correctly with a model trained with the data of testis but neither age effect nor age-tissue interaction were significant (p > 0.01) for these genes. This demonstrated that the overall gene expression levels during 15–60 days, in other words, the hidden patterns during 3–15 days, played a critical role in the model training and validation for these GO terms.

**Table 3:** Age and tissue effects on the expression of member genes involved in two predictable GO biological processes ^a^

**Term**	**Gene**	**Age effect**	**Tissue effect**	**interaction**
GO:0006122	CG17856	7.02E-01	3.79E-04	6.25E-01
GO:0006122	CG3560	7.68E-02	1.51E-08	6.49E-01
GO:0006122	CG3731	3.27E-03	1.29E-19	4.07E-01
GO:0006122	CG4169	3.73E-01	2.11E-08	9.99E-01
GO:0006122	CG4769	7.62E-01	2.44E-12	2.63E-01
GO:0006122	CG7580	2.51E-03	7.41E-14	6.29E-01
				
GO:0016579	CG12082	6.56E-02	3.96E-08	4.89E-02
GO:0016579	CG1490	1.44E-01	1.46E-01	1.99E-01
GO:0016579	CG15817	7.27E-01	5.67E-05	4.70E-01
GO:0016579	CG4165	6.35E-01	8.09E-05	6.57E-01
GO:0016579	CG5384	1.09E-01	4.81E-07	5.55E-02
GO:0016579	CG5798	9.07E-01	4.66E-01	9.76E-01
GO:0016579	CG7023	9.52E-01	1.26E-01	9.55E-01
GO:0016579	CG8494	8.97E-01	2.44E-02	9.19E-01
GO:0016579	CG8830	4.64E-02	8.38E-04	7.04E-01

^a ^listed in 3–5^th ^columns are the p-values.

## Discussion and Conclusion

Genome sequencing has led to the discovery of tens of thousands of potential new genes. Determining their functions seems far from a trivial task. One crucial constraint is the difficult in generating useful hypotheses about protein function [3]. Temporal microarray gene expression data is a valuable source for generating hypotheses about protein function. In this study, we predicted the GO biological processes based on multiple-tissues transcriptional profiles of aging in flies. Compared with Lagreid et al (2006) [6], our work included more GO terms, especially those highly specific biological processes which were not represented in the literature. More important, the prediction was based on the transcriptional profiles of aging rather than of the response to an artificial treatment, thus can provide insight into the genetic base of aging. In addition to 21 term-tissue specific models that had middle to high accuracy and will be helpful in detecting the genes involved in the corresponding biological processed, another three results from this study may be important.

Firstly, we proposed the concept of term-tissue specific prediction models. That is, given a biological process, the favorite prediction models may be trained on the base of the gene expression data sourced from a special tissue although it is possible that the combined data will lead to better classification. A little extension of the concept is "term-condition specific model." Based on the verification, this extension would be instructional for developing special DNA microarray experiments with the prediction of gene function as the main objective. The reason is that, in one condition or natural process of aging, only a small part of expressed genes have significant patterns, thus the number of predictable biological processes are very limited as shown in Lagreid et al (2006) and the current work. But the integration of data generated in multiple conditions will represent a richer information source.

The second finding was that the memberships of the genes involved in all the three children biological processes (in our annotated data set) on mitochondrial electron transport can be predicted from the transcriptional profiles of aging. This is interesting because the genes of mitochondria play a critical role in the longevity of C. elegans and D. melanogaster [18-21]. Another reason is that the genes involved in these biological processed also are members of some GO cellular components and GO molecular function related with mitochondria. For examples, the six genes in GO: 0006122 also are in GO terms mitochondrial respiratory chain complex III (cellular component) and ubiquinol-cytocgrrome-c reductase activity (molecular function).

The third finding was the hidden gene expression pattern of aging, which sourced from the design of the microarray experiments generating the analyzed data and represented a valuable information source for the functional prediction of new genes. It may be a pitfall in significant test and should be given severe attention.

Although genes that constitute a GO term are biologically related, their corresponding temporal expression profiles can be very different including, for instance, inverse co-regulation or co-regulation with a time lag or a combination of both. In this context, Lagreid et al's (2006) rule-based methods should be more effective than the currently used SVM method. But the former method does not fit our datasets because the small number of time points. On the other hand, our future effort will be focused on developing a hybridized algorithm based on unsupervised clustering algorithms and SVM to address the problems from inverse co-regulation or co-regulation with a time lag and integrate the data sourced from multiple tissues.

As described in the last section of result part, a GO term may be predicted using the transcriptional profiles even when the time effect was not significant for the member genes. But this was not the only case fitting all GO terms. For some predictable terms, the time effects were highly significant. Time series data is necessary for GO predictions, even for the terms in table 3. The prediction power mainly sourced from the difference between the individual term and the others. Suppose the genes in term A do not have significant time effects and the average expression level across the time frame is X. Again, suppose the genes of other terms with the average expression levels close to (or different from) X have significant time effects. In this case, Term A will have a different pattern from others and, therefore, can be predictable.

It is worthy to be noted that although the major part of the pursued GO biological processes had very low sensitivity or/and specificity in this paper, it does not means they can not be predicted. It is possible that some of them can be predicted by using the gene expression data of other tissues beyond those used in this study. Furthermore, we can expect to have better results if there were more time points or more replicates in the data. On the other hand, some downstream GO terms had few genes in our list so that we could not get significant prediction models for them even they may be predictable in fact. This means that more term-tissue specific models are possible if a better data set containing more annotated genes will be available.

## Methods

### Data

Seven types of tissue or organs, namely accessory gland (ACG), testis, brain, gut, malpighian tubule (MT), dorsal thoracic muscle (DTM), and abdominal fat body were dissected out of male flies (*Drosophila melanogaster*) at age of 3, 15, 20, 30, 45 and 60 days old. Tissue (or organs) samples from four males of the same age were pooled together and used for RNA sample preparation. The reference RNA was prepared from the corresponding tissues of 3-day old flies and the expression profiles at each of the five age-points from 15 to 60 days were measured twice by using independently prepared duplicated samples. Two to four micrograms of experiment and reference RNA were used to generate cDNA for labeling with fluorescent dye Cy3 and Cy5, respectively. Hybridized slides were scanned with an Axon GenePix Scanner (Axon Instruments, Sunnyvale, CA). Raw microarray data was normalized with LOWESS followed by between-slide scaling using Median Absolute Deviation (MAD) method [22]. The genes with more than 3 missing values in the duplicated 5-time course samples for each tissue were excluded from further analysis and the remaining missing values were imputed using k-nearest neighbor algorithm [23]. After removing the repeatedly spotted genes, 5557 genes were remained for Gene Ontology annotation.

### GO annotation

According to the definition, a GO term may be any one of a biological process, a cellular component, or a molecular function. But our work was conducted only on the biological processes, and, hereafter, GO terms only represent biological processes. Using the tool NETAFFX query with Drosopila_2 as the option of GeneChip Array [24], we (after some technical steps) annotated the genes in our list to the most specific GO terms.

Of the filtered list containing over five thousands of elements, 1318 genes were annotated with at least one (2.8 as the average) GO terms for each one. The numbers of genes involved in each GO term ranged from 1 to 150. Hierarchical structure widely existed among the GO terms containing the same gene. Among the 312 GO biological processes to which the 1318 genes were annotated, 148 terms each contained at least 4 genes and were pursued in this paper.

### Statistical analysis

Although the same gene may be the member of two or more biological processes, the functional classification of an uncharacterized gene, namely the prediction of the membership of the gene in a special biological process, can be completed with binary classification methods. Here, we used Support Vector Machines (SVM) [25] by running function *svm *in a R package called *e1071 *[26,27] to train models. The quality was assessed via ten-fold cross validation. In the application, gene expression ratios at the five time points were input as the features. Based the memberships from GO annotation, the genes in the training set were divided into two classes, one labeled with the ID of a special GO term (positive class), and the other one labeled with a fake ID, such as "GO: 00000000". In literature, Brown et al (2001) [5] used the method to predict five functional classes from the Munich Information Center for Protein Sequences Yeast Genome Database (MYGD) [28]. Our primary analysis showed that the classification was insensitive to the choice of kernels. The results reported here came from using radical-kernel SVM with the cost parameter *C *(see the equation (4)) assigned as 1 (the default of the software). Most GO biological processes contained very few members relative to the total number of genes in the data. This led to an imbalance in the number of positive and negative training examples that, in combination with noise in the data, is likely to cause incorrect classifications. By assigning weights for the positive and negative classes with a heuristic technique (see next sect for detail), we got the "optimized" results that balanced the sensitivity (SN) and specificity (SP) for class prediction. SN is calculated as TP/(TP +FN) where TP (true positives) is the number of genes classified and annotated to the process and FN (false negatives) is the number of genes annotated but not classified to it. SP is calculated as TN/(TN+ FP), where TN (true negatives) is the number of genes neither annotated nor classified to the process and FP (false positives) is the number of genes classified but not annotated to it. One-tail Fisher's exact test was conducted on the all the cross validations and multiple testing was addressed using BH FDR procedure [10].

### Support vector machines (SVM)

Suppose we are given a set of labeled training data,

(1) **(x_1_, y_i_), (x_2_, y_2_),......(x_m_, y_m_) **⊂ *R*^*m *^× {±1},

SVM separates the different classes by a hyperplane

(2) ⟨**w, Φ(x)**⟩ + *b *= 0

corresponding to the decision function

(3) *f*(**x**) = *sign*(⟨**w, Φ(x)⟩ **+ *b*).

SVM uses an implicit mapping **Φ **of the input data into a high-dimensional feature space defined by a kernel function **k(x, x') **which returns the inner product ⟨**Φ(x), Φ(x')**⟩ between the images of two data points **x **and **x' **in the feature space. In the case of soft margin classification the primal optimization problem takes the form:

(4) $$\text{Minimize t}(w,\xi )=\frac{1}{2}{\left\|w\right\|}^{2}+\omega_{+}\text{C}\sum_{\text{i}\in \text{A}}\xi_{\text{i}}+\omega_{-}\text{C}\sum_{\text{i}\notin {\text{A}}_{-}}\xi_{\text{i}}$$

(5) $$\text{Subject to }\begin{array}{l} {\text{y}}_{\text{i}}\langle w,\Phi (x_{i})\rangle +\text{b}\geq 1-\xi_{\text{i}}\\ \begin{array}{cc} \text{i}=(1,2......\text{m}), & \xi_{\text{i}}>0, \end{array} \end{array}$$

where the slack variable *ξ*_*i *_measures the degree of misclassification of the datum ***x*_*i*_**, C is the cost parameter [25,26], and *ω*_+ _and *ω*_- _are the weights for positive and negative classes, respectively. SVM solution ***w ***has an expansion $w=\sum_{i}\alpha_{i}\Phi (x_{i})$ in terms of a subset of training substances that lie on the margin. Non-zero coefficients (support vectors) occur when a point (**x_i_**, *y*_*i*_) meets the constraint. The coefficients *α*_*i *_are found by solving a quadratic programming problem.

As mentioned in the last section, the assignment of class weights *ω*_+ _and *ω*_- _is critical in the implementation for highly unbalanced data. In this study, we assigned the parameter with following formula:

(6) $$\begin{array}{ccc} \omega_{+}=\alpha \frac{\text{m}-{\text{m}}_{\text{A}}}{\text{m}} & \text{and} & \omega_{-}=\frac{{\text{m}}_{\text{A}}}{\text{m}} \end{array}$$

where m is the number of total instances (genes) in the training set, m_A _is the numbers of instances of the positive class A (a special GO term to be separated from others), and the tuning parameter *α *was optimized as 0.85 via a primary cross validation.

## Authors' contributions

WZ carried out statistical analysis and drafted manuscript. SZ carried out molecular genetic studies and participated in writing. JS supervised the analysis and participated in writing. All authors contributed to the design of the project. All authors read and approved the final manuscript.
